# Real-World Implementation of Particle-Based Microfluidics: On-Spot Test for Iron and Copper Ions in Water

**DOI:** 10.1021/acsomega.4c10152

**Published:** 2025-01-06

**Authors:** Indrek Saar, Hanno Evard

**Affiliations:** Chair of Analytical Chemistry, Institute of Chemistry, University of Tartu, Ravila 14a, 50411Tartu, Estonia

## Abstract

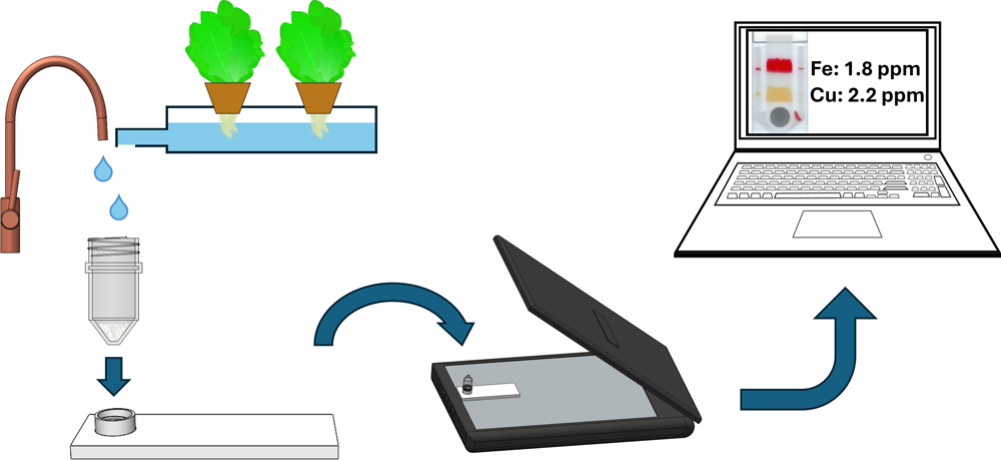

Water is an essential part of everyday life, and similarly, numerous industries depend on it. Regular water analysis is needed for both home use and in more specific fields, e.g., in agriculture, to ensure necessary quality. For an inexpensive and more convenient alternative to laboratory analysis, reliable and easy-to-use on-spot analysis devices are required. In this study, a novel particle-based microfluidic test for the quantitative analysis of iron and copper ions was developed. This is the first time that particle-based microfluidics has been demonstrated in a self-contained test to analyze real-world samples. The working range for both analytes was from 0.05 to 5 ppm with detection limits of 38 ppb for copper and 10 ppb for iron. This was achieved through a novel approach with sequentially placed immobile colorimetric reagents that can concentrate the analytes from the sample. In addition to the excellent suitability of monitoring drinking water, the applicability of the test to samples containing chelating agents was demonstrated. Different tap water samples and nutrient solutions from a hydroponic farm were used to successfully cross-validate tests’ performance with accredited laboratory methods. To the authors’ knowledge, such on-spot tests for complex samples containing strong chelating agents have not been successfully demonstrated before. Overall, the tests required under a minute of hands-on time, and no expertise is needed to perform the analysis. An ordinary portable flatbed scanner was used for quantification, allowing the entire analysis to be performed on the spot.

## 1 Introduction

Monitoring the iron and copper ion content in water is crucial for various applications, as both metals can have a significant impact on water quality. Iron levels are reportedly on the rise throughout Europe and North America.^1^ The recommended iron limit by the World Health Organization (WHO) in drinking water is 0.3 ppm, however, this level can be up to several orders of magnitude higher.^2^ In addition to potential health risks,^3^ a high iron content is undesirable because of staining and damaging appliances over long exposure times.^4,5^ Copper concentrations can also reach several parts per million, especially in the case of copper plumbing. The maximum permissible concentration for copper by the WHO is 2 ppm. However, this level can be exceeded if the water remains in pipes for long periods.^2^ Similar to iron, a high copper concentration can have a negative impact on water quality (e.g., bitterness in taste for drinking water and corrosion in boiler systems) and increase health risks.^5,6^

On the other hand, both iron and copper ions are important micronutrients, and monitoring their levels in agriculture is crucial to achieve higher yields and avoid over- and under-fertilization.^7,8^ This is especially critical in the case of hydroponics, i.e., soilless culture systems, where growth rates are faster and the crops are regularly exposed to the nutrient solution.^9,10^ The iron concentrations in the nutrient solution usually vary in the range of one to several ppm, whereas for copper they remain mostly below 0.1 ppm.^9,11^ However, this is greatly influenced by the crops, their growth stage, the specifics of the system, and even the location of the sampling site in the system, demonstrating the need for regular monitoring.^10^ The metal ions are often added as chelates to improve the stability of the nutrient solutions.^8,12^ Moreover, the use of chelating agents is common in many other applications, e.g., detergents, paper production, or in food industry, which also means that the compounds reach afterward to the natural environment.^13,14^ However, this makes the analysis of metal ions significantly more difficult with the simple spectrophotometric methods because the chelating agents interfere with the complex formation between the metal and the colorimetric reagent, causing errors in results.

As an alternative to spectrophotometry, a highly accurate and sensitive determination can be achieved using inductively coupled plasma (ICP) optical emission spectrometry (OES) or mass spectrometry (MS).^15^ However, expensive instruments and qualified specialists are needed to perform the analysis. Moreover, collection and transportation of the samples to the laboratory are required, which can be costly and time-consuming. To ensure that the concentrations of the metals remain in the required range, less costly and simpler monitoring methods must be used. For instance, several on-the-spot chemical analysis devices have been developed for transition metals analysis.^16,17^ Among them, microfluidic paper-based analytical devices (μPADs) are especially promising because of their portability, low cost, and ease of operation.^18,19^ Furthermore, when combined with colorimetric detection, they are also easy and intuitive to use. For signal readout, simple-to-use and widely available devices, such as smartphones^20−23^ or scanners^24,25^ can be used. Alternatively, completely instrument-free signal readout has also been demonstrated (e.g., with distance-based μPADs)^26−28^; however, only semiquantitative analysis can be performed with them, and reading the results is highly susceptible to user bias.

Although there are numerous promising demonstrations in laboratory settings, μPADs have still not managed to reach commercial use. It has been previously suggested that such practical focus is crucial when developing novel microfluidics technologies aimed at solving real-world problems.^29,30^ Our group has developed a novel type of capillary-driven microfluidics that uses porous particle-based materials.^31−33^ Similar to μPADs, passive liquid transport is used, which eliminates the need for external pumps and maintains simplicity and a low cost of analysis. The advantage of particle-based microfluidics (PBM) is the option to use particles of different sizes, porosities, and surface chemistries instead of paper to produce whole chips or specific areas of chips. This way, better control over the material flow characteristics and analytical functionality can be achieved, which leads to developing devices that are specifically suited for the desired analytes or processes from the ground up. Moreover, the selection of commercially available particles is significantly broader compared to that of paper. Combining paper-based materials with PBM is also possible and might be preferred for some regions (e.g., sample addition and waste areas). All of these aspects can significantly increase the analytical performance of the technology. Finally, as the PBM chip is formed using screen printing, its entire manufacturing process consists of only one step, making it suitable for fast prototyping while also being scalable for mass production.

The goal of this research article is to demonstrate for the first time that PBM can be used to create a practical, simple-to-use test that can be used to analyze real-world samples. For this, a simple colorimetric test for the quantitative analysis of total dissolved iron and copper in water samples is demonstrated. Moreover, for real-world use, the test should be able to measure the analytes from real samples at the relevant concentration levels (0.1–5 ppm) with less than 10% relative standard deviation (RSD) and complete the entire analysis within an hour. Such high performance has not yet been demonstrated with PBM. To achieve the set targets, several novel aspects were incorporated into PBM. First, immobilized colorimetric complexing reagents are used which can concentrate the analyte ions from a large amount of sample, leading to high sensitivity. Second, compared to similar distance-based μPADs,^27,34^ this is the first time that two complexing reagents are placed sequentially. This increases the selectivity of the test and allows the use of the entire sample more efficiently. Third, using the particle-based material instead of paper, allowed to omit the use of secondary reagents often needed to make the combination of a substrate and the colorimetric reagent compatible for analysis.^20,24,25^ Due to these aspects, a unique test with high sensitivity and a wide working range was achieved that is ideal for real-world applications. Lastly, a novel approach was used to diminish interference from strong chelating agents by adding large amounts of competing metal cations (e.g., Al(III)) to the sample. Due to this addition, to the authors’ knowledge, this is the first time that iron and copper could be measured from complex hydroponics nutrient water with on-spot colorimetric tests. For signal readout, flatbed scanners were used to avoid issues with variability in lighting conditions and differences between smartphone models.

## 2 Experimental Section

### 2.1 Chemicals

Bathophenanthroline (BP) (Sigma-Aldrich, 97%) was used for iron detection and bathocuproine (BC) (BLDpharm, 98%) was used for copper detection. In the sample addition tube, l-ascorbic acid (Roth, >99%), l-ascorbic acid sodium salt (Thermo Scientific, 99%), potassium alum dodecahydrate (Sigma-Aldrich, >98%), and sodium chloride (Lach:ner, 99.5%) were used. Iron(II)sulfate heptahydrate (Thermo Scientific, 99.5%), copper(II)nitrate trihydrate (Sigma-Aldrich, 99–104%), and iron(III)nitrate nonahydrate (Sigma-Aldrich, >98%) were used to prepare analyte standard solutions. Throughout this work, the analyte metals are mostly referred to as simply iron and copper instead of their specific ionic forms. After preconditioning the sample, most of the dissolved iron ions will exist as Fe(II) and copper ions as Cu(I), which then allows us to determine their total concentration in the sample. Concentrated hydrochloric acid (10 μL per 10 mL) was added to the 1000 ppm iron stock solutions. Fresh dilutions were prepared for all validation measurements and mostly a mixture of copper and iron (100 or 10 ppm) was used, which was diluted to the final concentration.

For selectivity measurements, nitrate salts of Cr(III), Co(II), Mn(II), Ni(II) Zn(II), and magnesium sulfate and calcium chloride were used. Diethylenetriaminepentaacetic acid (DTPA), ethylenediamine-N,N′-bis(2-hydroxyphenyl)acetic acid (EDDHA), N,N′-bis(2-hydroxybenzyl) ethylenediamine-N,N′-diacetic acid (HBED) monohydrochloride, a disodium salt of ethylenediaminetetraacetic acid (EDTA), and sodium citrate (CIT) were used for studying the influence of chelating agents. In the case of the chelating agents in acidic form, a 10 mM solution was first prepared in 0.1 M NaOH. Then, a 1 mM solution of every chelating agent was prepared into sodium acetate buffer (prepared from glacial acetic acid and sodium acetate trihydrate with pH = 5) with the final acetate concentration of 50 mM. In the mixture with iron and copper solution, the concentration of chelating agents was 35 μM, which was slightly above the total analytes’ concentration.

Deionized water from a Milli-Q system (Merck Millipore) was used for all experiments, and all chemicals used were at least reagent grade.

### 2.2 Screen Printing the Microfluidic Chip

The microfluidics chip part of the test (xanthan gum-bound silica gel particles on a glass substrate) was made with screen printing. The used equipment and general protocol are described in our previous work.^31^ The slurry for printing consisted of 470 mg silica gel particles (LiChroprep 15–25 μm) per 1 mL of xanthan gum (Sigma-Aldrich) solution (prepared in deionized water with 4 mg mL^–1^ concentration). A screen with T48 polyester mesh (Seritek OÜ), with a mesh opening of 142 μm, was used for printing, and 76 × 26 mm glass microscope slides (VWR) were used as the substrate. Four layers were consecutively printed on top of each other, and the resulting printed material thickness varied between 220 to 270 μm. The thicknesses were determined after drying, using a digital YATO YT-72305 micrometer with 2 μm accuracy.

The silica gel particles were washed before use since they (as well as all other available silica gel particles in the suitable size range) contained a considerable amount of iron. The silica particles were washed several times with hydrochloric acid and sodium ascorbate, followed by deionized water and drying at 110°. For more details, see ESI S1. Furthermore, some residual iron remained even after washing, and therefore even tests with blank samples had a low background signal for iron.

### 2.3 Test Design and Preparation

**Figure 1:**
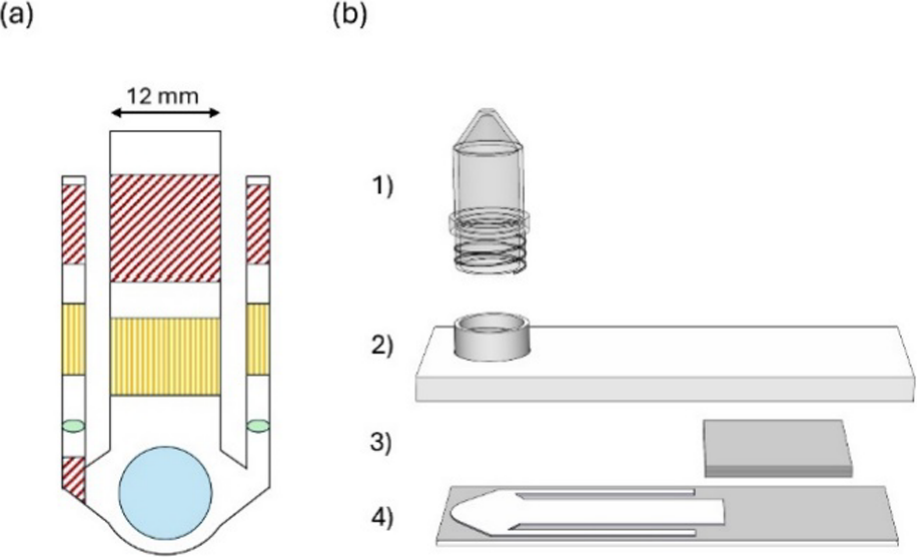
Test design and components. (a) Design and different regions on the printed chip. BP areas are indicated with red diagonal lines, and BC areas with orange vertical lines. Sample addition area is shown with a blue circle, and the control solution areas are indicated with green ellipses. (b) Exploded view of the assembled test. Components: (1) centrifuge tube for sample addition; (2) 3D-printed cover with a threaded insert; (3) stacked paper adsorption pads; (4) screen-printed chip on a microscope slide. The clamp to force the cover into contact with the printed material is not displayed.

The screen-printed chip consisted of one main channel (12 mm width) and two narrow side channels (2.5 mm width) acting as control channels (Figure 1a). The main channel was further connected from the top with four stacked paper pad pieces (Whatman CF4 paper with dimensions of 2.5 × 2.4 mm) to allow all of the samples to flow through the test. The sample addition area was at the bottom end of the chip at the connection of the three channels.

To prepare the chips for analysis, fixed amounts of colorimetric reagent solutions were pipetted onto specific areas on them. Twenty-four microliters of BP solution (2.6 mg mL^–1^ in ethanol) were added to the main channel, 4 μL for the top part of the control channels and 2.5 μL for the connection region of one of the controls. Eighteen microliters of BC solution (2.2 mg mL^–1^ in a 15% (v/v) mixture of chloroform in ethanol) were pipetted onto the main channel and 2.5 μL to the control channels. Approximately, a 4 mm gap was kept between the two reagent areas, and at least a 5 mm gap was left between the main BC region and the sample addition area. The reagents were pipetted under a closed cover next to an open container of the solvent to limit evaporation during the addition process. After both reagents had been added to the chips, they were dried in an oven at 90 °C for 10 min. Finally, 1 μL of control solution (10 ppm mixture of Fe(II) and Cu(II)) was added to both control channels, and the chips were left to dry at room temperature before assembling the test.

### 2.4 Assembled Test and Performing the Analysis

The assembled test (Figure 1b) consisted of the analysis chip, paper adsorbent pads, and a 3D-printed cover. As a separate piece, the cover had a threaded insert for connecting the centrifuge tube. The chip and cover were fastened with transparent tape at the absorbent pads and with a 3D-printed clamp at the sample tube. A good contact between both the printed chip and paper pads as well as the chip and the threaded insert was required to ensure optimal working of the test. An Ender 5 Pro 3D printer with arctic white polylactic acid filament (Spectrum Filaments) was used to produce all the 3D printed parts. SolidWorks 2023 engineering and Creality Slicer 4.8.2 software were used for designing all the elements.

Micro screw cap centrifuge tubes (0.5 mL with 10 mm outer diameter, Nerbe Plus) were used for adding the sample to the test. The tubes were prefilled with a salt mixture of ascorbic acid (0.048 M), sodium ascorbate (0.102 M), potassium alum (0.01 M), and sodium chloride (0.02 M) with the estimated concentrations for 400 μL of sample in the brackets. A larger portion of these salts was ground into a homogeneous mixture with a mortar and pestle, and approximately 13.8 mg was weighed into each tube. The pH of the mixture in deionized water was measured to be 4.2.

To perform the analysis, 400 μL of the sample was pipetted into the centrifuge tube, and the salt mixture was allowed to dissolve. After 15 min, the tube was screwed into the threaded insert of the test, and the test was turned around to let the sample come into contact with the chip. Next, an opening was punctured with a needle into the top of the sample tube to avoid a pressure drop inside. The running time of the test was approximately 35–40 min (until no excess liquid was seen on the sample addition area). Then, the 3D-printed clamp was removed, and the test was placed on a flatbed scanner for imaging.

### 2.5 Signal Detection and Quantification

Three tabletop flatbed scanners: Plustek OpticSlim 550 Plus (PLUS), Epson Perfection V39II (EV39), and Epson Perfection V600 (EV600) were tested for signal quantification. All automatic regimes and color balancing were turned off to avoid any bias and overcompensation by the scanner. Moreover, the test on the scanner bed was covered with a cardboard box to avoid any influence from external light. All images were stored in JPEG format and scanned at 600 dpi. ImageJ image processing software was used to quantify the signals from all of the images. More detailed information about the used calculation algorithms is presented in ESI S2.

## 3 Results and Discussion

### 3.1 Buffer Salt Mixture

To guarantee uniform conditioning of the entire sample and compensate for potential variances with real samples (e.g., in pH and temperature), simple pretreatment took place in a centrifuge tube before adding the sample to the test. If the preconditioning components were present in the porous material in the dry state, only a small initial portion of the sample would dissolve the components, and they would not interact with the remainder of the sample. Moreover, because both BP and BC are oxidation state-specific, forming a detectable complex with only Fe(II) and Cu(I), respectively, allowing sufficient time for the reduction reaction to complete is paramount. When choosing a suitable reducing agent, long-term stability, low toxicity, and environmental friendliness were specially considered to make the tests viable for mass production and wide use. Among the commonly used reducing agents for similar applications,^35−37^ ascorbic acid fulfilled all these criteria. In a mixture with sodium ascorbate, buffering also provided the benefit of avoiding precipitation of the analytes during pretreatment and ensuring their mobility on the silica gel. The buffer concentration (0.15 M) was chosen to be relatively high (over 1000 times molar excess to the analyte concentrations in the upper limit of the working range) to compensate for any potential variance in the sample matrix while maximizing the reduction of analytes.

Fe(II) was used to calibrate the test. Therefore, for estimating Fe(III) in the sample, the reduction efficiency of Fe(III) to Fe(II) was studied. This was necessary since Fe(II) is often prevalent in water samples where the dissolved oxygen content is low (e.g., groundwater), but it is converted to Fe(III) in samples with higher oxygen content (e.g., surface water). However, in the case of unknown samples, the ratio between the two can vary, and if only Fe(III) is used for calibration of the developed test, the iron content can be overestimated. The reducing efficiency was determined by measuring Fe(II) and Fe(III) solutions at the same concentration. A statistically significant (*p* = 0.05) 11% decrease in intensity was seen with Fe(III) indicating incomplete reduction. However, in the case of the real samples, no significant difference was seen with the reference measurements. For measuring copper, the test was calibrated with Cu(II). The reducing efficiency of Cu(II) to Cu(I) was not separately studied because copper ions primarily exist in aqueous solutions in Cu(II) form. Therefore, even if the reduction is not complete, the determined calibration curve would already account for it in the working range.

Sodium chloride was added to the sample tubes to improve the sensitivity of copper detection. This has been previously reported for similar systems and is mostly attributed to the stabilizing effect of chloride on cuprous ions.^25,38^ Finally, the mixture contained potassium alum to compete with the analyte ions for binding to the potential chelating agents present in the sample. Aluminum ions were chosen as they form relatively strong bonds with many chelating agents,^39^ while they differ markedly from the analyte ions so that interfering with the detection is avoided. Performing the sample pretreatment in the centrifuge tube prior to adding it onto the chip gives time for the aluminum ions to replace the analyte in the interfering chelating agents.

### 3.2 Test Working Principle and General Aspects

The working principle of the test was similar to some previously reported distance-based μPADs,^26,40^ where immobilized complexing reagents (bound to the surface or simply insoluble in the sample solution) will capture analyte metals and form colored complexes. However, in the test reported here, a larger amount of sample (400 μL) was allowed to flow through the short reagent areas on a single wide channel, which essentially acted as solid-phase extraction regions for the analytes. This allowed positioning the reagents in series on the same channel, which makes the working principle also similar to affinity chromatography as different analytes are selectively captured and separated from the sample. Furthermore, both analytes could be detected from the same part of the sample as opposed to multiple separate channels for each analyte as usually demonstrated with μPADs.^20,24,26^ This placement resulted also in higher selectivity for iron detection without the use of masking agents (see discussion in Section 3.5). In addition, the stronger cation exchange properties of silica gel surface (when compared to paper) meant that no additional polymers were needed to keep the formed positively charged complexes from moving.^20,25,41^ Similarly, the strongly hydrophilic nature of silica gel ensured the uniform aqueous sample flow through the relatively hydrophobic colorimetric reagent areas without additional reagents.^24,25^

Although the entire analysis takes approximately 1 h to complete, the hands-on time for the analysis is under a minute. Moreover, the initial color response can be already seen in a minute in case of high analyte concentrations (e.g., immediately warning of high pollution in drinking water). However, in most cases, one hour for accurate quantitative results with high sensitivity is fit-for-purpose and excellent when compared to laboratory analysis. Furthermore, although an automatic pipet was currently used for adding the sample, this can be easily replaced by a disposable exact volume transfer pipet if measurements are performed outside of the laboratory. The relatively high sample amount was also chosen to minimize small errors in the sample addition while ensuring the availability of transfer pipettes with the corresponding volumes.

Two control channels were included in the test to visually confirm that all reagents were functioning, and the sample flow was controlled (e.g., if the test was flooded with the sample, the signals would strongly decrease). Moreover, control channels are crucial for verifying the long-term stability of the tests. Currently, storage for only 2 weeks and one month in a sealed vacuum bag was tested. Throughout this time, no statistically significant deterioration in the performance was observed. Furthermore, one control had an extra BP region to capture the analyte ions before reaching the other colorimetric reagent areas. This provided relatively constant signals on the control channel (i.e., signal only from analyte added to the test), while the other channel had a mixed response with the analytes from the sample and the analyte added to the test. The signal values for the constant control channel were also calculated, and they provided a correction factor for different imaging devices. With further investigation, a comparison of the control channel values can provide an estimate of the sample matrix effects. See ESI Figure S3 for more information.

### 3.3 Comparison of Scanners

For ease of use, it would be desirable that any scanner available for the analyst could be used for quantification of the signal. To determine whether different scanners could be used, tests for the calibration series were performed with three different scanners. The initial determined intensity values and image comparison can be seen in ESI Figure S4.

**Figure 2:**
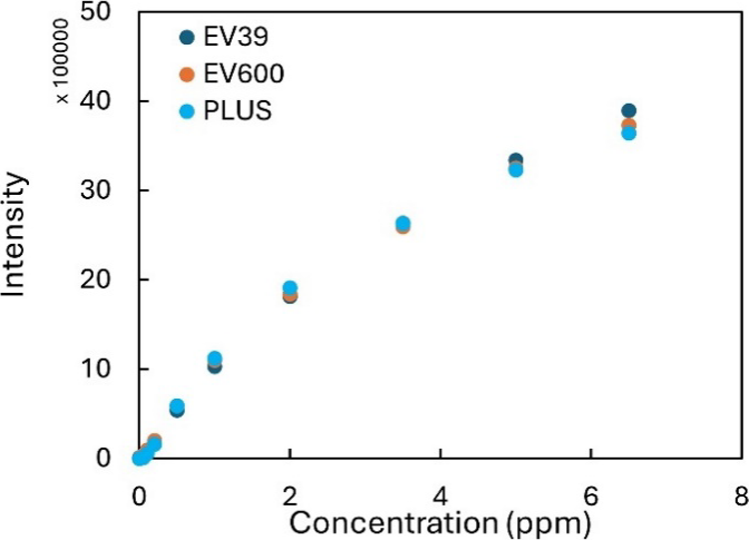
Comparison of different scanners for copper after applying correction factors based on the control signal. Initial results and corresponding data for iron can be seen in ESI Figure S4.

Although all scanners provided similar trends, the values varied significantly, indicating the need to calibrate the test separately for different scanners. Therefore, a correction factor was applied to the images from different devices. This is demonstrated in Figure 2, where the mean control value from each scanner was used to normalize the results, which significantly decreased the variance of the results between the scanners. A similar improvement was observed for iron when a correction factor was applied (see ESI Figure S4). It must be noted that the factors for iron and copper were different. Therefore, control areas for both analytes are needed to estimate the factor.

In conclusion, different scanners can be used for the signal readout. By applying correction factors, the variance between scanners is significantly decreased. For all of the subsequent experiments, the Epson Perfection V39II scanner was chosen because it provided the highest sensitivity. Moreover, this scanner is lightweight and portable, as it is powered by the USB interface of the computer that it is connected to, thus, no external power outlet is required, ensuring usability even for remote on-site analysis.

### 3.4 Limit of Detection and Linear Range

**Figure 3:**
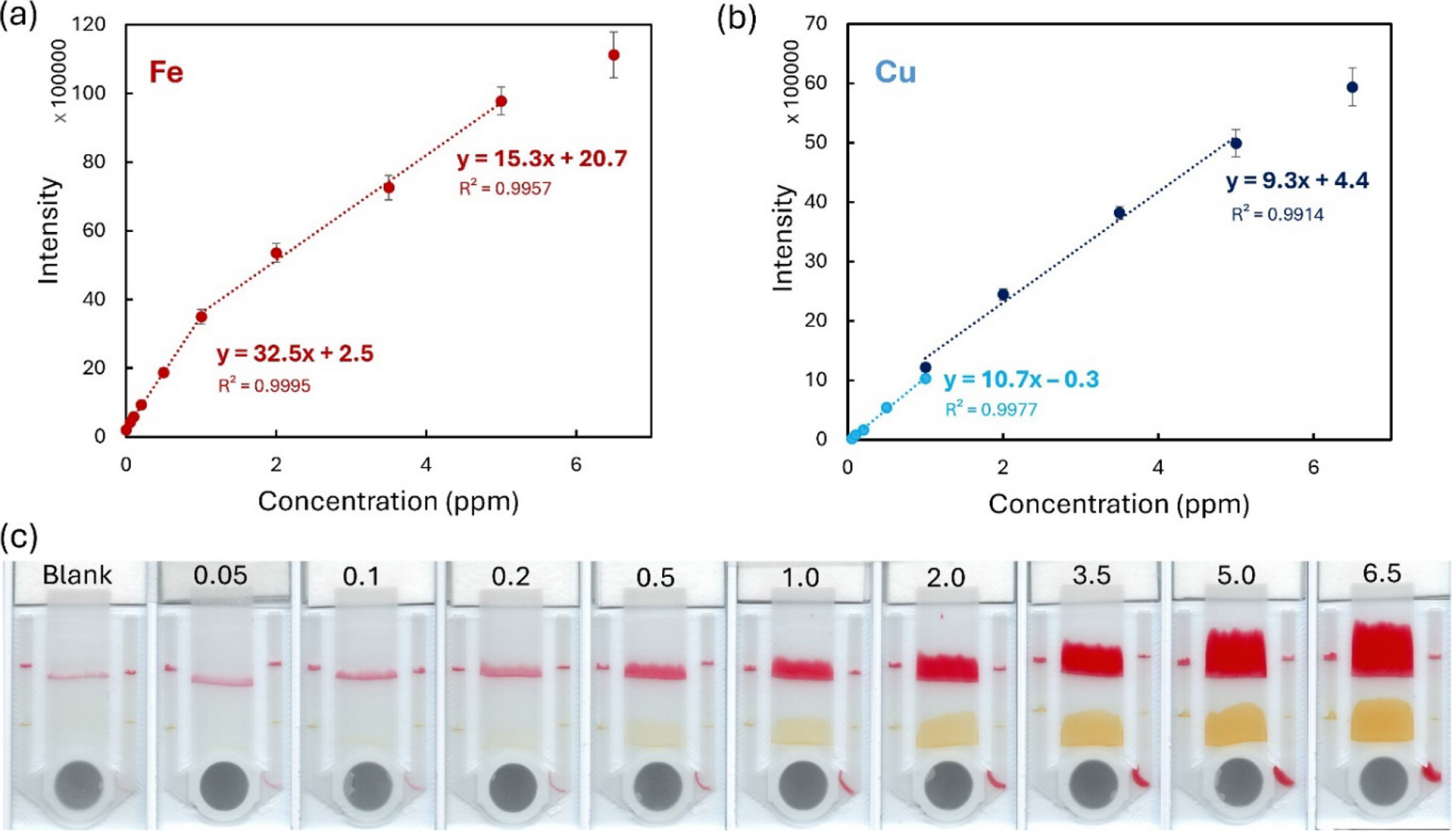
Calibration curves for (a) iron and (b) copper with the developed test (*n* = 5). Error bars show ± standard deviation (SD), within-lab reproducibility. The equations correspond to the presented *y*-axis values (they must be multiplied by 100,000 for calculation). (c) Scanned images of the tests in one calibration series. Analyte concentrations in each test are presented in ppm.

The recorded calibration curves are presented in Figure 3. Both analytes could be quantified over the entire investigated range of 0.05 to 6.5 ppm. However, two calibration regions were needed so that linear regression could be used. In the lower concentration range (below 1 ppm), the intensity of the color changed. In the higher concentration range (over 1 ppm), the signal area increased, and the color remained essentially the same (Figure 3c). This was especially prevalent in the case of iron but was also apparent in copper. Moreover, to ensure higher sensitivity in the lower range and better accuracy in the higher range, two different image processing algorithms were used for copper (see ESI S2 for more details). Two different intensity values are given at 1 ppm because the different image processing gives somewhat different intensity values for the calibration solution at 1 ppm (see Figure 3b). For quantification of samples with unknown concentrations, the lower range was used for intensities that corresponded up to 1 ppm (included), and for any higher intensity values, the higher range was used.

The limits of detection (LoD) for iron and copper were calculated to be 10 and 38 ppb, respectively. For more details on the estimation of the LoD, see ESI S3. The working range for both metals ranged from 0.05 to 5 ppm, with relative standard deviations between 4.1–7.8% for iron and 2.8–8.9% for copper. A discussion and comparison of these results to some of the previously published μPADs for the detection of iron and copper are provided in ESI S4 and Table S1. The test developed in this work provided high sensitivity and a wide working range simultaneously for both iron and copper. Crucially, the working range is exactly suitable for real-world applications for samples, such as drinking water, industrial water, and agriculture. These aspects are unique to the developed tests compared to the tests in the literature. Moreover, in the case that some specific applications demand higher sensitivity or wider working range, the test can be further modified for them. For example, a larger amount of sample can be used to achieve higher sensitivity or the colorimetric reagent concentration can be increased to raise the upper limit of the working range.

### 3.5 Selectivity

Both BP and BC were chosen because they are highly selective toward their corresponding analytes.^42,43^ Based on previous reports and initial testing,^44^ only Cu(I) gave a colored complex with BP in addition to Fe(II) under the current conditions. This interference was eliminated by placing the BC region before BP so that the Cu(I) ions are captured by BC and will not reach BP. However, since other transition metal ions have also been reported as potentially interfering compounds (e.g., by consuming the reagents), Zn(II), Cr(III), Mn(II), Ni(II), and Co(II) were chosen for further investigation.^43,44^ Their concentration was set to 0.1 ppm, which in the case of drinking water is higher than the established limit or expected value in most real samples. The corresponding analyte ion concentration was 0.5 ppm, which is in the middle of the working range as well as a reasonable concentration for potential real samples. In addition, higher concentrations (100 ppm) of calcium and magnesium were studied as possible interferents. Possible interference from sodium, potassium, and aluminum was not further investigated because they were already part of the buffer mixture.

**Figure 4:**
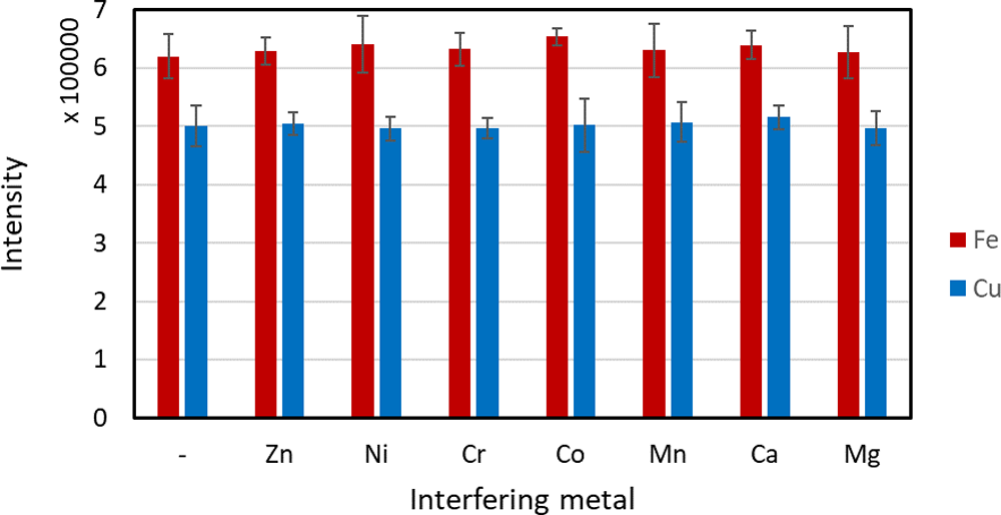
Results of the selectivity evaluation (*n* = 5). Error bars show ±SD, within-lab reproducibility. The two leftmost bars show the corresponding analyte signals without interfering ions. The concentrations of iron and copper were 0.5 ppm, and of all interfering transition metals, 0.1 ppm. The calcium and magnesium concentrations were 100 ppm. For better visualization in this graph, all of the values for iron are divided by three.

The results are presented in Figure 4. None of the selected metal ions significantly altered the test results at the studied concentrations. When MgCl_2_ was used instead of MgSO_4_, an increase of approximately 10% in the copper signal was observed. This can be attributed to the stabilizing influence of chloride ions on Cu(I) ions.^38^

### 3.6 Influence of Chelating Agents

**Figure 5:**
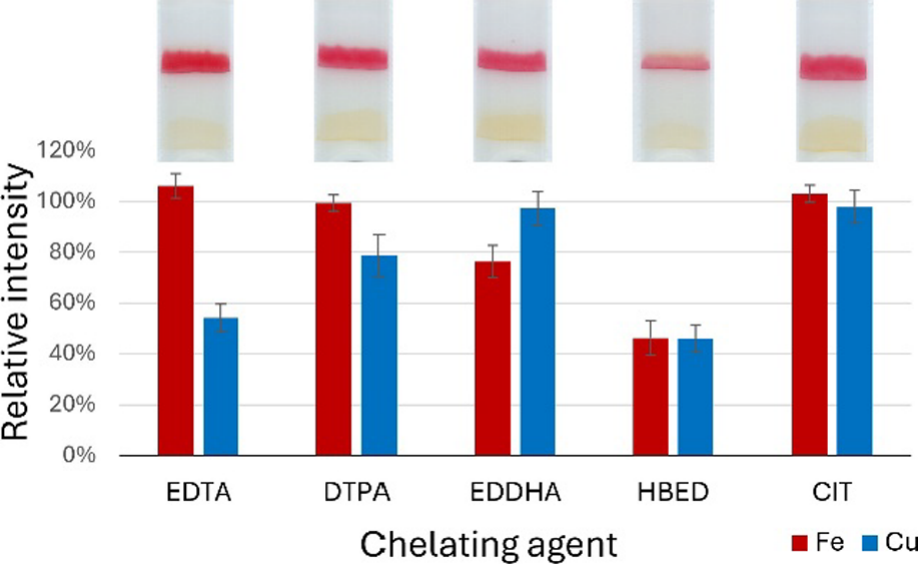
Influence of the chelating agents on the measured intensities (*n* = 4). Corresponding images of the tests are shown above the bar chart. Error bars represent the mean ± SD and within-lab reproducibility. The values obtained with chelating agents are divided by results of comparison measurements with a 1 ppm mixture of Fe(III) and Cu(II). The analyte concentration was 1 ppm, and the molar concentrations of the chelating agents were equivalent to the total molar analyte concentration.

To investigate the applicability of the test for different agricultural samples, where nutrients are used in chelated form, solutions with several chelating agents were prepared. While EDTA chelates of both iron and copper are common, DTPA, EDDHA, and HBED are mostly used for iron with increased strength in the named order.^45^ Furthermore, since iron occurs as Fe(III) in these chelates, iron(III)nitrate was used to prepare the solutions. In addition, sodium citrate was included because of its potential presence in real samples. The results are presented in Figure 5.

Different chelators had varying effects on the colorimetric complex formation. In the case of iron, a significant drop in the intensity of the signal was observed with EDDHA and HBED. For copper, EDTA and HBED had the strongest effect, while DTPA decreased the intensity of the signal by approximately 20%. Among the chosen chelators, HBED expectedly had the strongest influence on both metals, and it also led to mixed signals as copper was eluted over the BC region to the BP region, which led to the formation of the Cu(I)-BP complex (see ESI Figure S6b). This was also observed with EDTA, but the effect was significantly weaker. Therefore, in the case of lower copper concentrations, EDTA should not significantly affect the results for iron (as was the case with real samples from the hydroponic farm; see Section 3.7).

In all experiments, aluminum ions were added to the samples to replace the iron and copper in the chelates, making them available for detection. To estimate the efficiency of this strategy, comparison experiments were conducted using an aluminum-free buffer mixture. The results showed that the chelating agents reduced the signals even more strongly when alum was not present (e.g., for copper with EDTA and iron with HBED). Moreover, considerable elution of the colorimetric complexes started to occur, and with higher concentrations of analyte ions, this led to almost mixed signals. For more details, see ESI Figures S5 and S6.

In general, if the chelator is known, the developed test can be readily used with some chelating agents, and in the case of others, its influence on the signal can be compensated with an adjusting factor. For instance, with fertilizers used in hydroponics, the chelating agent information is usually provided on the package. Moreover, in follow-up experiments using indium(III) or gallium(III) instead of aluminum(III) ions, no significant drop in the signal for copper was observed with any chelating agent (ESI Figure S6). On the other hand, these metals had a negative effect on the BP complex, as it was significantly eluted and a loss of signal occurred. However, with further optimization of the used replacement ions mixture (e.g., a combination of aluminum and gallium) more universal compensation of chelating agents can be achieved.

### 3.7 Analysis of Real Samples

To validate the developed tests with real samples, drinking water with variable quality from five locations over Estonia was measured, including two sources with copper plumbing. For more complex matrices, six samples from a local hydroponic farm were collected from three different nutrient solutions and two separate locations in the system. These samples were more complex because they contained various other nutrient metals, chelating agents, as well as common anions used in fertilizers. For reference measurements, the samples were sent to an accredited laboratory (Estonian Environmental Research Centre), where they were analyzed according to the laboratory’s measurement protocol. The test results were compared with the reference measurements using the *E*_*n*_ parameter:
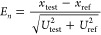
where the denominator is calculated using expanded uncertainties *U* (*k* = 2). In case |*E*_*n*_ | < 1, the agreement between results can be considered satisfactory. However, considering that the measurements were done on the same day in the case of the test developed in this article, the uncertainty values used are probably underestimated and the *E*_*n*_ values overestimated.

The results for drinking water are presented in Table 1. Most of the obtained values were in good agreement with the reference values and provided absolute *E*_*n*_ values of less than 1. Moreover, even for the values that were below LoD or the quantification limit (LoQ) of the developed test, the obtained reference values indicated that this semiquantitative estimate (i.e., <LoD or >LoD, <LoQ) was made correctly by the test. The only significant disagreement was in the case of sample DW5, which had the highest copper concentration. In DW5 the iron concentration was below the LoQ (but above the LoD). While the exact cause for this remains unclear, a possible interference from copper with the reference method used for that sample might have occurred. For the spectrophotometric method (ISO 6332), copper concentrations greater than 10 times can interfere with the results. This error does not occur in the case of the developed test because copper is bound to BC and cannot reach the BP area.

**Table 1:** Results with Drinking Water Samples (*n* = 3)

sample	metal	microfluidic test		reference method		*E*_*n*_
		value (ppm)	RSD^a^	value (ppm)	*U*^b^	
DW1	Fe	0.10	9%	0.12	12%	–0.87
	Cu	<0.038		0.0057	13%	
DW2	Fe	0.26	5%	0.24	12%	+0.53
	Cu	<0.038		0.0026	13%	
DW3	Fe	1.8	8%	1.8	12%	–0.06
	Cu	<0.038		0.00086	13%	
DW4	Fe	<0.010		<0.02	19%	
	Cu	0.12	14%	0.14	13%	–0.66
DW5	Fe	>0.010, <0.050		0.077	19%	
	Cu	1.1	4%	1.1	13%	–0.29

a Uncertainty values for the test measurements are provided as relative standard deviations (*k* = 1).

b Reference measurements for copper were done with ICP-MS (ISO 17294–2), and for iron with ICP-OES (ISO 11885) or spectrophotometrically (ISO 6332) for samples DW4 and DW5. The uncertainties were provided as extended uncertainty values (*k* = 2).

The results of the nutrient solution samples from the hydroponic farm are presented in Table 2. The results show that in most cases there is no significant variance between the results from the developed test and the reference method. One difference can be seen in the case of sample H1_1, as the test yielded an approximately 30% higher result for iron. The exact reasons for this are unclear, especially as it was expected that chelating agents might lead to a decrease in the intensity of the signals. Problems with sample handling may be a possible reason for this. However, a more thorough investigation is required to determine the exact causes. Copper was reliably detected with all of the microfluidic tests. However, the influence of the chelating agent was additionally considered, because it was known that EDTA chelate was used for copper. Therefore, an adjusting factor was applied to the results, assuming a 46% decrease in the intensity of the signal. Moreover, because of the influence from EDTA, all signals were near the detection or quantification limit, which also led to a large variance in the results.

**Table 2:** Results for Nutrient Solution Samples (*n* = 3)

sample	metal	microfluidic test		reference method		*E*_*n*_
		value (ppm)	RSD^a^	value (ppm)	*U*^b^	
H1_1	Fe	2.5	5%	1.9	12%	+1.77
	Cu	0.13	10%	0.14	8%	–0.49
H1_2	Fe	1.9	10%	2	12%	–0.17
	Cu	0.13	11%	0.16	8%	–0.81
H2_1	Fe	2.7	2%	2.4	12%	+0.97
	Cu	0.11	16%	0.14	8%	–0.71
H2_2	Fe	2.4	5%	2.5	12%	–0.23
	Cu	0.092	21%	0.1	8%	–0.20
H3_1	Fe	1.3	7%	1.4	12%	–0.21
	Cu	0.062	25%	0.055	8%	+0.22
H3_2	Fe	1.2	8%	1.2	12%	–0.03
	Cu	0.060	31%	0.048	8%	+0.32

a Uncertainty values for the test measurements are provided with relative standard deviation.

b Reference measurements were done with ICP-OES (ISO 11885) and their uncertainties were provided as extended uncertainty values.

In general, both types of samples could be accurately measured with the developed test and only two of the 22 obtained values differed significantly from the reference values. While the variance was quite significant in the case of copper in the nutrient solution samples, it could be improved by adding indium salt to the buffer mixture. Given the random sign of the differences between the values from the tests and the reference method, there appears to be no bias in the test results, in the case of either drinking water or nutrient solutions. It can therefore be concluded that the developed test is successful in the analysis of total dissolved copper and iron in very different water samples.

## 4 Conclusions

A particle-based microfluidics (PBM) test was developed for on-spot measurements of the dissolved iron and copper contents in water samples. Iron and copper were chosen as the analytes because of their wide occurrence in real-world samples. The working range for both analytes was 0.05 to 5 ppm, with a LoD of 38 ppb for copper and 10 ppb for iron. The test was simple to use (incl. the sample pretreatment phase) and could be operated on-spot, and only a lightweight portable scanner was used for quantification. These capabilities make the test ideal for quantitatively monitoring the levels of these metals in real-world situations. The analytical performance was achieved by applying a novel approach with PBM, where large amounts of sample could be eluted through areas with immobilized reagents that concentrate the analytes and give colorimetric signals. Detecting analytes in series further improved selectivity without using masking agents, and with silica gel as the base material, no polymers were needed to ensure the immobilization of the colorimetric reagents and their complexes with analytes. Therefore, the need for auxiliary reagents was reduced. In addition, the influence of different chelating agents was studied, and the possibility of quantifying iron and copper in hydroponics nutrient water was demonstrated by a novel solution of adding aluminum ions to the sample. Finally, all used chemicals and materials were inexpensive and environmentally friendly, and after use, the main test components (glass substrate, plastic cover) can be reused while the rest are recyclable. Therefore, it can be said that the goal often set, but seldom fully realized in microfluidics, to demonstrate a complete and potentially mass-producible analysis device that is suitable for regular consumer use, was fulfilled.

## Data Availability

The data supporting this article have been included as part of the Supporting Information.

## Supporting Information Available

Pretreatment of silica gel particles; ImageJ image processing algorithms; estimating the LoD and LoQ values; comparison with previously demonstrated μPADs; interpreting signals from control channels; scanner results comparison; influence of aluminum with chelating agents; and influence of using Al(III), Ga(III), In(III) with different chelating agents (PDF)
