# Supplementary material for: Real-World Implementation of Particle-Based Microfluidics: On-Spot Test for Iron and Copper Ions in Water
Source: ACS Omega. 2025 Jan 6;10(1):1800–8. doi: 10.1021/acsomega.4c10152 (PMC11740257; doi:10.1021/acsomega.4c10152)
Supplement: Supplementary file 1 — ao4c10152_si_001.pdf [file ao4c10152_si_001.pdf]

Electronic Supporting Information

Real-World Implementation of Particle-Based Microfluidics: On-Spot Test for Iron and Copper Ions in Water

Indrek Saar and **Hanno Evard***

Chair of Analytical Chemistry, Institute of Chemistry, University of Tartu, Ravila 14a, 50411 Tartu, Estonia
*E-mail: hanno.evard@ut.ee

S1 Pretreatment of the silica particles

Interference from iron was a substantial problem in the test development (similar issues for copper were not present). Silica gel particles from different manufacturers were tested, however they all gave considerable background signal if mixed with ascorbic acid and bathophenanthroline (Figure S1 a). Therefore, pretreatment of the particles was required before screen printing. A solution of hydrochloric acid (0.5 M) and sodium ascorbate (0.1 M) was poured onto the particles in a 50 ml falcon tube (with 30 ml of the solution per 6.6 mg of particles) and the tube was thoroughly shaken to reach a homogeneous mixture. It was then left on an orbital shaker for 24 hours, followed by replacing the solution with deionized water (25 ml) two times. To replace the solution, the tube was left standing vertically and when the particles had sedimented out, the previous solution was poured off and new one was added. In case of deionized water, the tube was simply thoroughly shaken and the particles were immediately allowed to sediment. This entire process was repeated 2 times (incl. the steps with HCl and ascorbate) and then the particles were left on the orbital shaker with a 0.15 M ascorbate buffer (pH = 4.5) overnight. Finally, the solution was replaced with deionized water six times before the particles were poured on a petri dish and left to dry in an oven for 12 hours at 110 degrees.

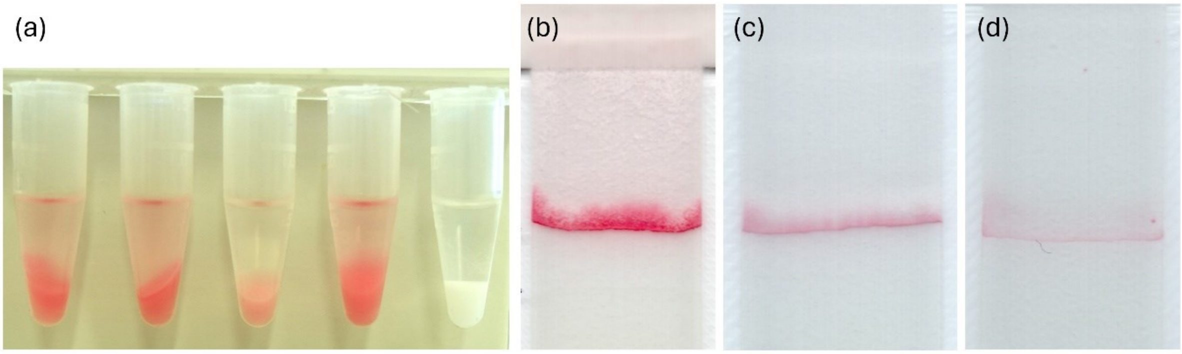

Figure S1. Pretreatment of silica gel particles. (a) Initial iron content in different silica gel particles, rightmost tube in the row contains washed particles. (b) Printed test channel with poorly washed particles, pink background is visible over the entire colorimetric reagent area. (c) Blank signal with initial washing protocol. (d) Blank signal with improved washing protocol (described above).

The particles were washed separately for each series of the printed chips and the results were verified with blank samples to determine the amount of background signal. The washing protocol also changed slightly throughout the experiments as initially lower reagent concentrations and less steps were performed. In Figure S1 b, weak background signal over the entire bathophenanthroline area can be seen, showing poor results of the washing. These particles were deemed unsuitable for use. In Figure

S1 c and d only signal at the edge of the colorimetric reagent area can be seen and both of these batches were deemed suitable. The background signal of each batch was also taken into account in calculations when the concentration value was under 0.5 ppm. At higher concentrations, the impact of the background signal was marginal (less than 5% of the total signal). For even better results, higher concentration of the washing solution components and an increased number of steps could be used.

S2 ImageJ image processing algorithms

To quantify the iron signals in ImageJ, a rectangular region of interest was drawn over the entire BP area, the three color channels of the image were split and the green channel values were subtracted from the red channel values. The choice of the color channels was based on the highest contrast between two channels throughout the entire calibration range. Next, an automatic threshold with Huang method was applied to the result to confine the signal area and then total pixel intensity was calculated. The analysis process for iron is illustrated in Figure S2.

In general, the same principles were applied for copper detection. However, two different algorithms were used depending on the concentration to provide better results. This was needed due to changes in the bathocuproine complex color tone, because with the tone the ratio between different color channels changed as well. At lower concentration range (0 – 1 ppm) and for control channels, blue channel values were subtracted from the green channel values. For higher concentrations (over 1 ppm), red channel was used instead of green in the calculations as it provided higher signals. Moreover, default thresholding method of ImageJ was applied on the blue channel, which was then combined with the subtracted values to confine the signals in the image with the subtracted value. The total pixel intensity was calculated to obtain the signal.

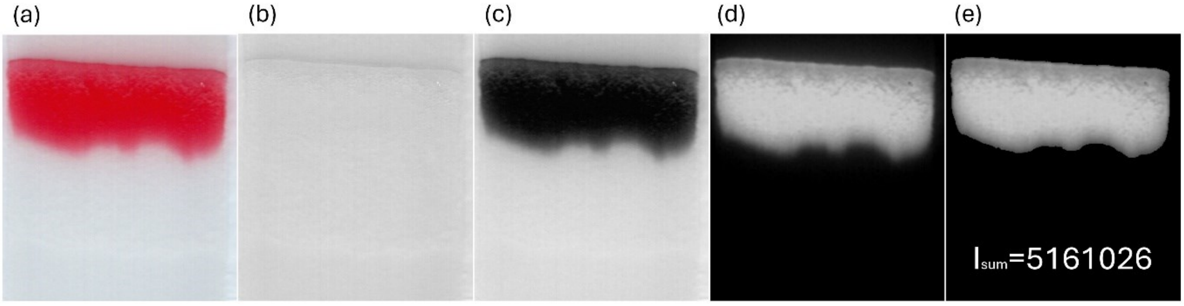

Figure S2. ImageJ analysis steps for iron (2 ppm). (a) Selection and cropping of the entire colorimetric reagent area; (b) Split red channel; (c) Split green channel; (d) Green channel has been subtracted from the red channel; (e) Threshold has been applied to the results and summative pixel intensity is calculated.

Total pixel intensity values of the formed complexes were used and preferred over mean values or areas of the formed complexes. This was done because slight variances in the printed material thickness and reagent concentration had less effect on the total intensity value (e.g. with thinner printed material, the complex area was larger but the maximum intensity was lowered). Similarly, if some component of the sample matrix (e.g. chelating agents or other cations) lead to elution of the colored analyte complex, total intensity value would be less influenced than, for example, the area of the colored complex.

S3 Estimating the LoD and LoQ values

The detection limits (LoD) were calculated using the equation:

$$LoD = \frac{(\bar{y}_o + 3.3 \times S(y)) - a}{b},$$

where $\bar{y}_o$ was the mean value of blank samples, $S(y)$ was the standard deviation of blank samples, a was the intercept and b the slope of the calibration curve.

For the limit of quantification (LoQ), the lowest measured concentration (0.05 ppm) was chosen for both analytes, as it was repeatedly possible to quantify that amount with suitable precision. For iron, lower values could be also obtained with additional experiments. The obtained LoD and LoQ values were also critically estimated, based on the real samples measurements and if there was a difference in the results compared to blank.

S4 Comparison with previously demonstrated µPADs

Many paper-based analytical devices (µPADs) have been proposed for the quantitative or semi-quantitative detection of iron and copper ions from different water samples. Table S1 contains some of the most relevant examples. Moreover, both single analyte and multiplexed systems, where up to 6 metals were detected simultaneously with the device are included. Before comparison, an important notion must be made regarding the oxidation state of the analyte metals. In many published works, clear reference is only made to investigating Fe(III) with phenanthroline-based reagents and reducing agents, which essentially allow to determine the total dissolved iron content. However, since the efficiency of the reduction reaction is usually less than 100% (especially with short reaction times), the developed test can incorrectly estimate the iron content in samples. This is also the reason, why sample pretreatment in separate tubes was applied in this work to ensure higher accuracy for different real samples.

Table S1. Comparison of the analytical characteristics with other developed µPADs for iron and copper.

Metal	Reagent	LoD (ppb)	Sensor range (ppm)	Signal readout device	Year	Reference
Cu(II)	sodium diethyldithiocarbamate	290	~0.29–20 [a]	Phone camera + controlled lighting	2014	Wang et al.[1]
Fe(II,III)<br>Cu(II)	Bathophenanthroline<br>Dithiooxamide	20000<br>100000	20–1300<br>100–1300	Visual, distance-based	2015	Cate et al.[2]
Fe(II,III)<br>Cu(II)	Bathophenanthroline<br>Zincon	100<br>100 (1) [b]	0.1–20<br>0.1–20	Visual, radial distance based	2018	Hofstetter et al.[3]
Cu(II)	1-(2-pyridylazo)-2-naphthol (PAN)	22 [b]	0.064–0.44	Scanner	2019	Wu et al.[4]
Fe(III)<br>Cu(II)	1,10-phenanthroline<br>Bathocuproine	200<br>30	0.3–18<br>0.05–24	Smartphone + controlled lighting	2019	Li et al.[5]
Fe(III)	1,10-Phenanthroline	5600	5.6–280	iPad camera	2020	Xiong et al.[6]
Cu(II)	Bathocuproine	320	0.32–63.55	Scanner	2021	Kamnoet et al.[7]

Fe(III)<br>Cu(II)	Bathophenanthroline<br>Bathocuproine	1100<br>300	0.5–15<br>0.1–5	Smartphone + lightbox	2023	Aryal et al.[8]
Fe(II,III)	Naphthalene-3-hydroxy-4-pyridione ligand	70	0.25–2	Scanner	2023	Aguiar et al.[9]
Cu(II)	rhodamine-based chelator	10	0.05–0.5	Scanner	2024	Aguiar et al.[10]
Fe(II,III)<br>Cu(II)	Bathophenanthroline<br>Bathocuproine	10<br>38	0.05–5<br>0.05–5	Scanner		This work

[a] Remains uncertain from the presented data.

[b] Achieved with a separate solid phase extraction system.

In general, our reported detection limits are among the lowest, although higher sensitivities especially for copper have been achieved. However, in several cases separate SPE system for preconcentration was used for that, which involves application of additional eluents by the user. In terms of the demonstrated sensor range, two orders of magnitude is among the widest ranges and the necessity to measure concentrations at even higher concentrations can be considered rare. Finally, using scanner for quantitative results is still the preferred choice by many researchers, since additional components and lighting control is required for detection with smartphones.

S5 Figures

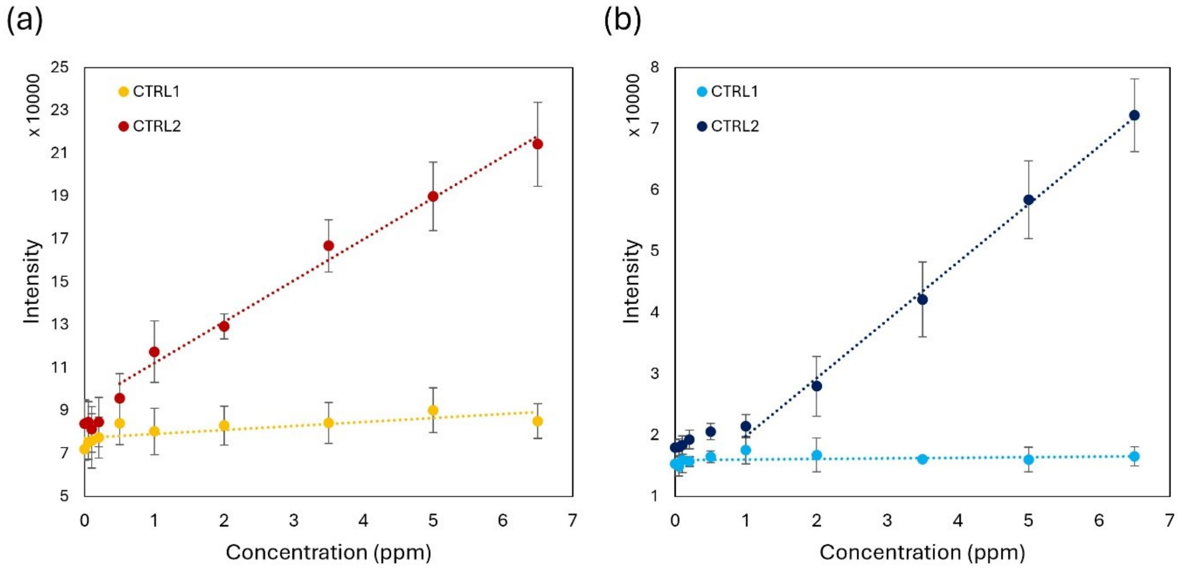

Figure S3. Interpreting signals from control channels (n = 5, error bars show +/- standard deviations). (a) Difference between the “constant” (CTRL1) and “mixed” (CTRL2) control signal values for iron. (b) Difference between the “constant” (CTRL1) and “mixed” (CTRL2) control signal values for copper. In case of both metal ions, CTRL1 values do not depend on the analyte ions concentration in the sample. Moreover, CTRL2 values could be used to estimate the analyte concentration in the sample and provide a comparison value to the main test channel in case they are above 1 ppm (i.e. if these two values match, this could provide an additional guarantee on the results).

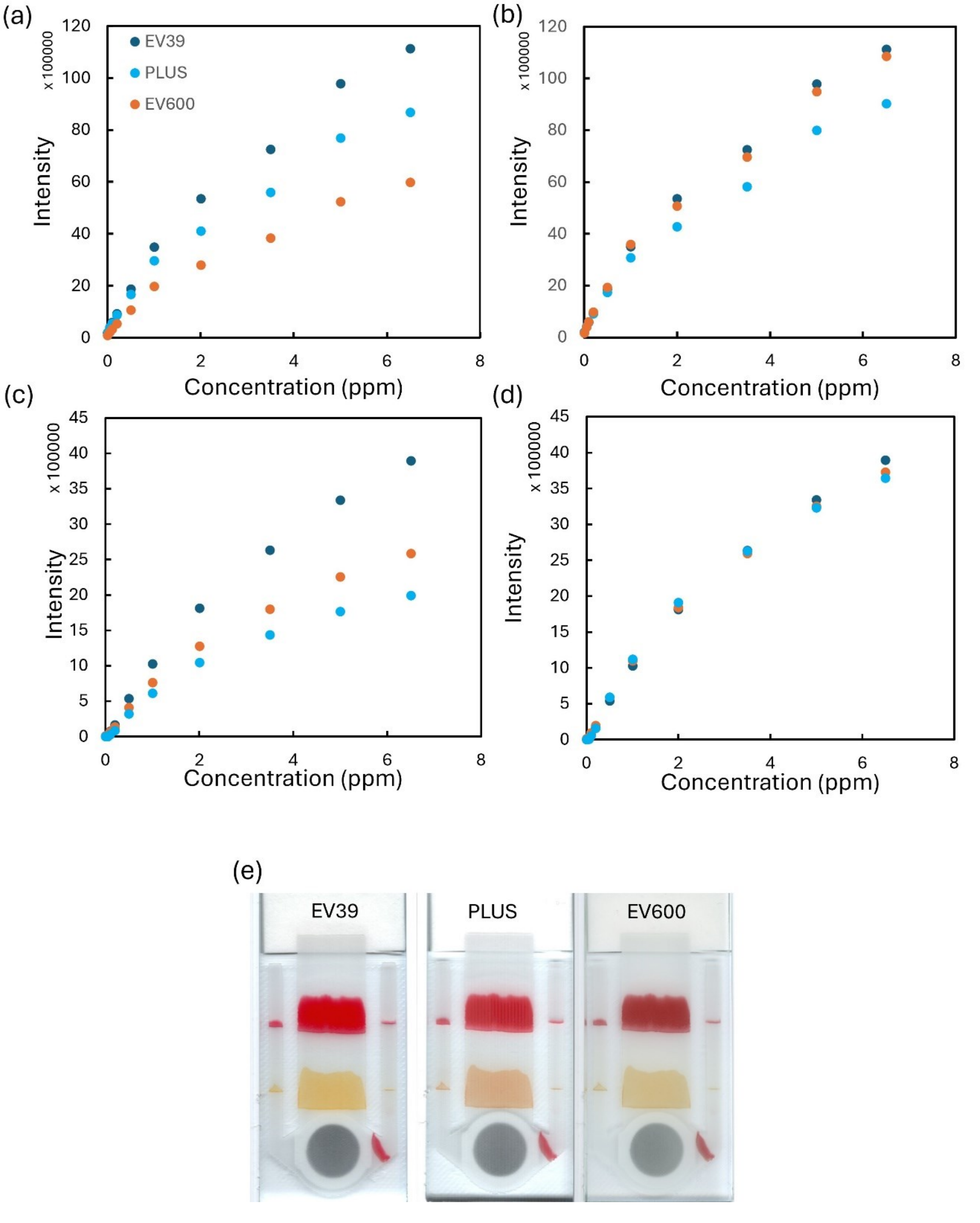

Figure S4. Scanner results comparison. (a) Initially obtained values for iron. Legend is same for all graphs. (b) Values after applying correction factor for iron. The reason for the deviation at higher concentrations for the Plustek scanner was estimated to be the lower color depth and the larger lateral pixel area. (c) Initially obtained values for copper. (d) Values after applying correction factor for copper. (e) Different scanner image comparison for 3.5 ppm analyte mixture.

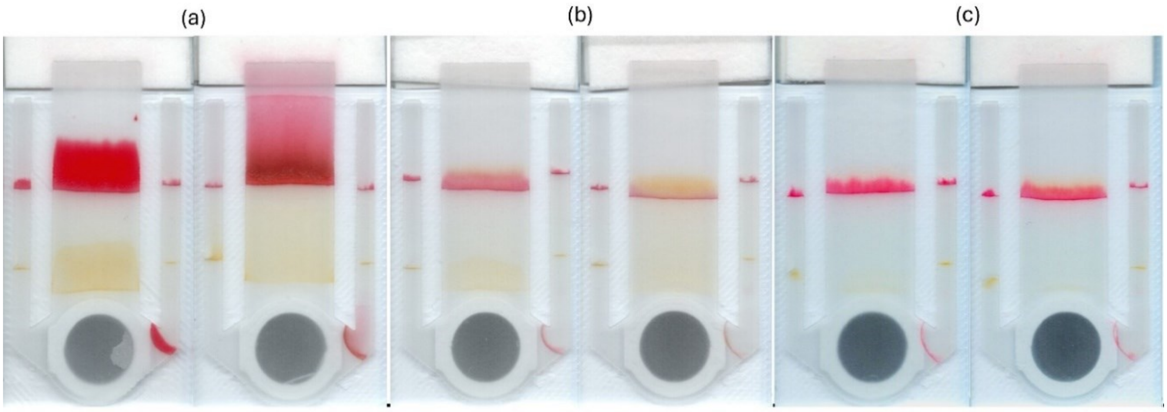

Figure S5. Influence of aluminum with chelating agents. (a) DTPA with 3.5 ppm analyte concentration with and without added aluminum. (b) HBED with 1 ppm analyte concentration with and without aluminum (c) EDTA with 0.2 ppm analyte concentration with and without added aluminum. With aluminum, signal at the BC region is stronger and without aluminum, more "golden" background can be seen at the BP region (these pictures are both slightly enhanced in the same way to better illustrate the difference).

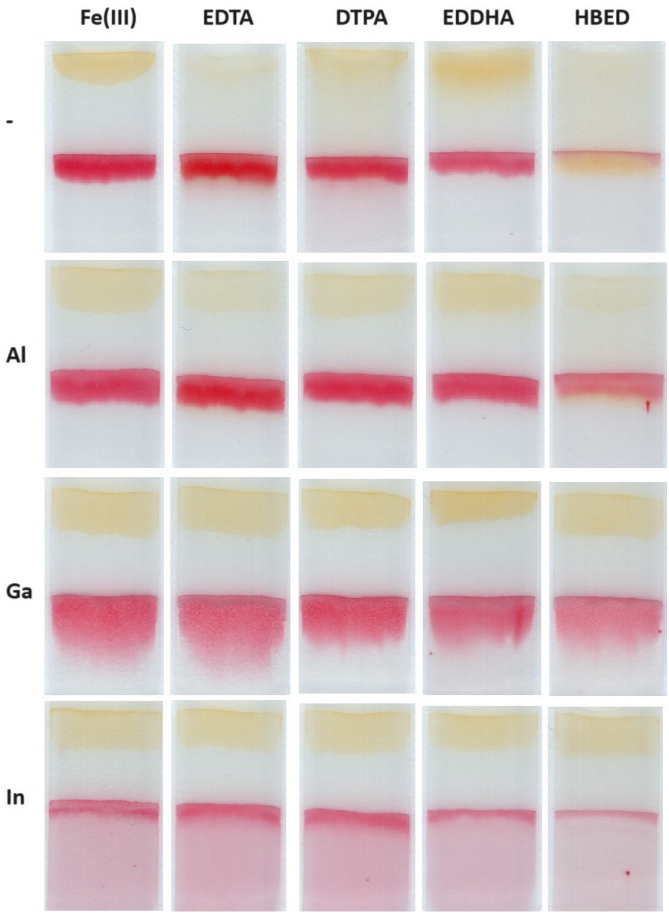

Figure S6. The influence of using Al(III), Ga(III), In(III) with different chelating agents. In the first row, no replacement ion was present in the sample tube. By using gallium or indium, the detection of copper is significantly improved compared to Al(III) and only a small variance in signal was observed. In case of iron, the spreading of the formed bathophenanthroline complex increases with the replacement ion size.

References

(1) Wang, H.; Li, Y.; Wei, J.; Xu, J.; Wang, Y.; Zheng, G. Paper-Based Three-Dimensional Microfluidic Device for Monitoring of Heavy Metals with a Camera Cell Phone. *Anal Bioanal Chem* **2014**, *406* (12), 2799–2807. https://doi.org/10.1007/s00216-014-7715-x.

(2) Cate, D. M.; Noblitt, S. D.; Volckens, J.; Henry, C. S. Multiplexed Paper Analytical Device for Quantification of Metals Using Distance-Based Detection. *Lab Chip* **2015**, *15* (13), 2808–2818. https://doi.org/10.1039/C5LC00364D.

(3) Hofstetter, J. C.; Wydallis, J. B.; Neymark, G.; Iii, T. H. R.; Harrington, J.; Henry, C. S. Quantitative Colorimetric Paper Analytical Devices Based on Radial Distance Measurements for Aqueous Metal Determination. *Analyst* **2018**, *143* (13), 3085–3090. https://doi.org/10.1039/C8AN00632F.

(4) Wu, Q.; He, J.; Meng, H.; Wang, Y.; Zhang, Y.; Li, H.; Feng, L. A Paper-Based Microfluidic Analytical Device Combined with Home-Made SPE Column for the Colorimetric Determination of Copper(II) Ion. *Talanta* **2019**, *204*, 518–524. https://doi.org/10.1016/j.talanta.2019.06.006.

(5) Li, F.; Hu, Y.; Li, Z.; Liu, J.; Guo, L.; He, J. Three-Dimensional Microfluidic Paper-Based Device for Multiplexed Colorimetric Detection of Six Metal Ions Combined with Use of a Smartphone. *Anal Bioanal Chem* **2019**, *411* (24), 6497–6508. https://doi.org/10.1007/s00216-019-02032-5.

(6) Xiong, X.; Zhang, J.; Wang, Z.; Liu, C.; Xiao, W.; Han, J.; Shi, Q. Simultaneous Multiplexed Detection of Protein and Metal Ions by a Colorimetric Microfluidic Paper-Based Analytical Device. *BioChip J* **2020**, *14* (4), 429–437. https://doi.org/10.1007/s13206-020-4407-9.

(7) Kamnoet, P.; Aeungmaitrepirom, W.; Menger, R. F.; Henry, C. S. Highly Selective Simultaneous Determination of Cu(II), Co(II), Ni(II), Hg(II), and Mn(II) in Water Samples Using Microfluidic Paper-Based Analytical Devices. *Analyst* **2021**, *146* (7), 2229–2239. https://doi.org/10.1039/D0AN02200D.

(8) Aryal, P.; Brack, E.; Alexander, T.; Henry, C. S. Capillary Flow-Driven Microfluidics Combined with a Paper Device for Fast User-Friendly Detection of Heavy Metals in Water. *Anal. Chem.* **2023**, *95* (13), 5820–5827. https://doi.org/10.1021/acs.analchem.3c00378.

(9) Aguiar, J. I. S.; Ribeiro, S. O.; Leite, A.; Rangel, M.; Rangel, A. O. S. S.; Mesquita, R. B. R. Iron Determination in Natural Waters Using a Synthesised 3-Hydroxy-4-Pyridione Ligand in a Newly Developed Microfluidic Paper-Based Device. *Chemosensors* **2023**, *11* (2), 101. https://doi.org/10.3390/chemosensors11020101.

(10) Aguiar, J. I. S.; Ribeiro, S. O.; Leite, A.; Rangel, M.; Rangel, A. O. S. S.; Mesquita, R. B. R. Use of a Rhodamine-Based Chelator in a Microfluidic Paper-Based Analytical Device for the *in-Situ* Copper Quantification in Natural Waters. *Talanta* **2024**, *271*, 125683. https://doi.org/10.1016/j.talanta.2024.125683.
